# An Audit to Assess Nicotine Management on a Mental Health Rehabilitation Unit in Mersey Care NHS Foundation Trust

**DOI:** 10.1192/bjo.2024.541

**Published:** 2024-08-01

**Authors:** Ranjan Baruah, Olusegun Popoola, Sion Gilbey, Oliver Pentz

**Affiliations:** Mersey Care NHS Foundation Trust, Liverpool, United Kingdom

## Abstract

**Aims:**

Research has found that having a mental health condition is associated with smoking, and difficulties remaining abstinent. It is also evidenced that there is desire to reduce the amount smoked and cease smoking altogether by those with mental health conditions. Smoking can also affect some medications used to treat mental health conditions.

To assess nicotine replacement management in inpatients at Rathbone Rehabilitation Centre (RRC) against Mersey Care NHS foundation Trust (MCFT) Nicotine Management Guidelines (SA20).

**Methods:**

Data of all discharged patients from RRC over a 12-month period was collected following a standardised process and assessed for 6 parameters.

A total of 51 discharges were identified and the whole sample of 51 patients were audited.

**Results:**

47 (92%) were asked and recorded of their smoking status and 4 (8%) were not at the point of first contact on patient electronic records (Rio).

Of the 28 smokers who were identified on admission, 26 (93%) were offered support to stop smoking at that point. 3 other patients started smoking during admission.

Of the 31 patients who were identified as smokers (including 3 who began smoking during admission), 24 (77%) were offered support to stop smoking at regular intervals throughout their admission and 7(23%) were not.

Of the 28 smokers who did not wish to permanently stop smoking, there was documented evidence that 20 (71%) of these individuals were offered nicotine replacement treatment (NRT) in some form to manage temporary abstinence from smoking.

5 out of 31 smokers were referred to a Nicotine Dependence Treatment Advisor for counselling and support during their inpatient stay.

**Conclusion:**

Below action plan was designed to improve compliance with MCFT Nicotine Management Guidelines (SA20):

Audit leads to communicate with every team member at RRC (Team meetings and emails) to remind them of the following:

1. To offer smokers support to stop smoking at regular intervals and document on Rio; via named nurse sessions or opportunistically.
2. To offer NRT where appropriate and document on Rio if accepted or declined during MDT reviews/named nurse session.
3. Ensure Physical Health Nursing Proforma is always completed on Rio, and if the service user is a smoker, to ensure referral status (referred/declined) to Nicotine management team is documented.
4. Increase awareness of referral pathway by putting up posters in relevant clinical areas.

